# Computed tomography and pathological findings of five nasal neurilemmomas

**DOI:** 10.1186/1758-3284-4-26

**Published:** 2012-05-23

**Authors:** Jing Hu, Yang-Yang Bao, Ke-Jia Cheng, Shui-Hong Zhou, Ling-Xiang Ruan, Zhou-Jun Zheng

**Affiliations:** 1 Department of Otolaryngology, The First Affiliated Hospital, College of Medicine, Zhejiang University, Hangzhou, 310003, China; 2 Department of Radiology. The First Affiliated Hospital, College of Medicine, Zhejiang University, Hangzhou, 310003, China; 3 Department of pathology, The First Affiliated Hospital, College of Medicine, Zhejiang University, Hangzhou, 310003, China

**Keywords:** Neurilemmoma, Tomography, X ray computed, Pathology, Nasal cavity, Nasal sinus

## Abstract

**Objectives:**

Neurilemmomas are benign tumors deriving from Schwann cells of the nerve sheath. They occur in all parts of the body. The highest incidence of neurilemmoma is in the head and neck region (38–45%), but involvement of the nose and paranasal sinus is quite rare, with only sporadic cases having been reported in the world literature. Fewer than 4% of these tumors involve the nasal cavity and paranasal sinuses. We describe the clinical, pathologic, and computed tomography (CT) features of five nasal neurilemmomas.

**Methodology:**

CT features of five patients with nasal schwannoma proved by operation and pathology were investigated.

**Results:**

Schwannomas tend to be solitary and are usually well-circumscribed tumors with an oval, round or fusiform shape in the unilateral nasal cavity. The lesions usually have a mottled central lucency with peripheral intensification on contrast-enhanced CT scans. The heterogeneous appearance is related to areas of increased vascularity with adjacent non-enhancing cystic or necrotic regions.

**Conclusions:**

Schwannoma should be considered in the differential of unusual nasal masses. Certain clinical and CT patterns may be of use in the differential diagnosis.

## Background

Neurilemmomas are benign tumors deriving from Schwann cells of the nerve sheath. They occur in all parts of the body. The highest incidence of neurilemmoma is in the head and neck region (38–45%) [1,2], but involvement of the nose and paranasal sinus is quite rare, with only sporadic cases having been reported in the world literature. Fewer than 4% of these tumors involve the nasal cavity and paranasal sinuses [3].

The clinical symptomatology of nasal neurilemmomas is varied and nonspecific. The signs and symptoms usually depend upon the location or size of the tumor and subsequent involvement of surrounding structures. Preoperative diagnosis is facilitated by endoscopy, CT, and magnetic resonance imaging (MRI) [1-6]. CT reveals a unilateral nasal mass that may be expansile [6]. Some features of CT is helpful in differentiating neurilemmomas from malignancies. Schwannomas can cause bone remodeling by pressure and this behavior can lead to misdiagnosis as a malignant process [6]. Preoperative correct judgement can aid to perform appropriate surgical approach [1]. To our knowledge, there are few reports more than five cases of CT features of nasal schwannoma . In this study, we describe the clinical, pathologic, and CT features of five nasal neurilemmomas.

## Materials and methods

From March 2001 to Deotcember 2011, five cases of nasal neurilemmomas were surgically removed and pathologically confirmed at our hospital. The study was performed with approval of the institutional review board of institution. Informed consent was not required. We retrospectively reviewed the CT, clinical manifestations and pathological findings.

The routine CT studies, with and without intravenous contrast agent injection, were performed with contiguous 3.2 mm sections from the anterior edge of frontal sinus to the posterior edge of sphenoid sinus. CT findings were analyzed by three radiologists. All histologic slides were reviewed independently by two pathologists. The clinical data were obtained from an archive. Immunohistochemistry was performed using a streptavidin-peroxidase (SP) immunohistochemical staining technique. The primary antibodies consisted of S100 protein (polyclonal, dilution 1:300; Dako, Trappes, France).

Pathologic specimens, CT findings and clinical histories were reviewed.

## Results

### Summary of clinical findings in five cases of nasal neurilemmomas (Table 1)

**Table 1:** Clinical findings in five cases of nasal neurilemmomas

**Pt**	**Age(years)/Sex**	**Location**	**Duration of symptoms**	**Symptoms**	**Treatment**	**Malignant**	**Follow-up**	**Recurrence**
1	59/M	NV (right)	3 years	NO	S	No	12.8years	No
2	27/F	NV(right)	2 years	NO	S	No	12.7 years	No
3	51/M	NS(left)	10 years	NO, nasal bleeding, headache, rhinorrhea anosmia	S	No	12.3years	No
4	48/F	NS(right)	6 months	NO, nasal bleeding	S + R	No	4.8 years, died of heart diseas	No
5	56/M	NS (left)	2 months	NO, little nasal bleeding	S	No	7.8 years	No

*M* male, *F* female, *NC* nasal cavity, *NV* nasal vestibule, *NO* nasal obstruction, *S* Surgery, *R* Radiotherapy, *NS* Nasal Septum.

There were two women and three men, 27 ~ 59 years old. The mean age was 48.4 years. The history of symptoms was from two months to ten years. Clinical symptoms contributing to the diagnosis were varied and nonspecific, including epistaxis (1 case), unilateral nasal obstruction (4 cases). There were no incidences of tumor recurrence during the study period. The mean follow-up was 10.1 years (range 4.8–12.8 years).

### The results of rhinoscopy and nasal endoscopy ,surgery ,CT findings and pathology(Table 2, Figures 1,2,3,4,5,6)

**Table 2:** The results of rhinoscopy and nasal endoscopy , surgery ,CT findings and pathology

**Pt**	**rhinoscopy and nasal endoscopy**	**Findings during surgery**	**CT findings**	**pathology**
1	A smooth mass in right nasal vestibule and diffuse swelling of right nasal alar and also left deviation of the columella. The tumor was tough and painless.	The tumor was completely removed via a gingivobuccal incision under local anesthesia. The tumor at right nasal vestibule was firm, ovoid, smooth, encapsulated.	CT imaging revealed that a well-defined ovoid soft tissue mass without central in the right nasal vestibule. The mass of density was uneven. The mass had an attenuation number of 17Hounsfield units (Hu) to 43Hu.On contrast CT, there was mild enhancement(20Hu~50Hu) The nasal sinuses were clear (Figure 1).	Grossly, the tumor was round, smooth, encapsulated. On cut surface, the mass was grayish, spiral and some areas was cystic. Microscopically, the tumor cells were composed of spindle cells arranged in fascicles. The tumor cells had a wavy shape, poorly defined cytoplasm, and oval nuclei with tapering ends. S-100 positive (Figure 2).
2	A firm, fixed oval mass about 1.8 cm × 1.5 cm × 1.0 cm in infer-internal of left nasal vestibule, very close to the nasal valve angle and columella was deviated to right.	The tumor was completely removed via a gingivobuccal incision under local anesthesia. The mass at left nasal vestibule was firm, round, smooth, encapsulated.	CT imaging showed that a well-defined round soft tissue mass without central in the right nasal vestibule. The mass of density was uneven(20 ~ 36Hu). There was patchy enhancement on contrast CT. The nasal sinuses were clear (Figure 3).	Grossly, the tumor was round, smooth, encapsulated. On cut surface, the mass was grayish, spiral and some areas was cystic. Microscopically, the tumor cells were composed of spindle cells arranged in fascicles with some thickened hyalinized vessels. No evidence of vascular thrombosis was observed. S-100 positive.
3	The left nasal cavity was full of lobular, light yellow mass with yellowish bleeding secretion on the surface. The nasal septum was deviated to right and was adhesion to the right inferior turbinate.	A 5.0cm × 6.0cm tumor was completely removed via lateral rhinotomy. Its pedicle was attached to the nasal septum. The external lateral wall of the left nasal was partial absorbed and the left maxillary sinus was involved.	CT scan showed that a well-defined large expansile soft-tissue mass in the left nasal cavity from choana to the inferior turbinate, which extended up to the left maxillary sinus with evidence of bony dehiscence of the internal lateral wall. The nasal septum was deviated to right. There was patchy enhancement after iodinated contrast administration, which CT value was 20Hu-41.2Hu. In addition, soft tissue density was noted in the left ethmoid sinus, compatible with sinusitis due to obstruction of the sinus orifice by the mass (Figure 4).	Grossly, the tumor was irregular, soft, unencapsulated. On cut surface, the mass was composed of gray firm tissue with a large central cystic space with yellow colored inner surface. Microscopically, A spindle cell neoplasm, with hypercellular areas and edematous hypocellular areas. Palisading nuclei consistent with Verocay bodies. S-100 strong positive.
4	A smooth tumor about 3.0 cm × 2.0cm located at the posterior left surface of nasal septum. The nasal common meatus had some pink nasal discharge.	A3.0cm × 2.0cm tumor was completely removed via lateral rhinotomy. The mass was limit to the left nasal cavity and from nasal septum.	CT scanning showed a 2.0 cm in diameter soft density mass that filling the left choana and extended into the nasopharynx. The partial bone of the nasal septum was absorbed. The left maxillary had soft tissue density compatible with sinusitis due to obstruction of the sinus orifice by the mass. CT with contrast injection showed that the mass enhanceing inhomogeneously (Figure 5).	On gross examination, the mass was encapsulated. It measured 3.0cm × 2.0cm × 2.1cm, and it had a rubbery consistency. Microscopic examination revealed spindle cells with indistinct cell outlines and a moderate amount of cytoplasm. S-100 protein immunostaining was positive.
5	A smooth, pedunculated, gray mass covering with blood crust was in the top of left surface of nasal septum.	Endoscopic sinus surgery was carried out. The mass was about 1.0cm × 1.0cm in the anterior top of the left septum. The tumor excised completely with a safemargin of surrounding normal septal mucoperiosteum.	A well-defined, inhomogeneous, 1.0 cm × 1.0cm soft mass that located at the anterior of the left nasal vestibule was observed by CT scan. The mass had an attenuation number of 17 (Hu). The bilateral frontal, sphenoid nasal sinuses were clear and the bilateral maxillary and ethmoid sinuses were lower density due to obstruction of the sinus orifice by the mass. There was homogeneous enhancement on contrast CT(50Hu) (Figure 6).	Grossly, the tumor was round, smooth, encapsulated. On cut surface, the mass was grayish, spiral and some areas was cystic. A Pathological examination shows high cellular density and palisading pattern.The tumor cells are immunoreactive for S-100 protein.

**Figure 1:**
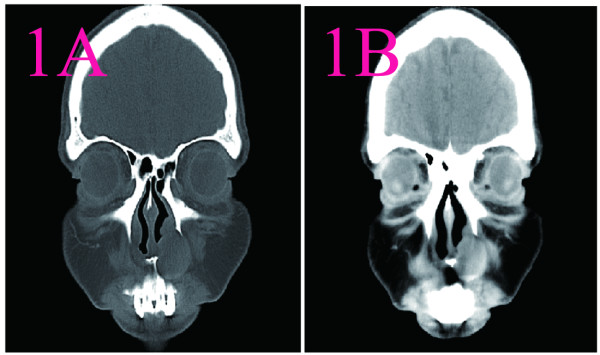
**CT scans of nasal cavity in Case 1.A**: plain scan: a well-defined ovoid soft tissue mass without central in the right nasal vestibule. The mass of density was uneven(17Hu~43Hu). **B**: On contrast CT, there was mild enhancement(20Hu~50Hu).

**Figure 2:**
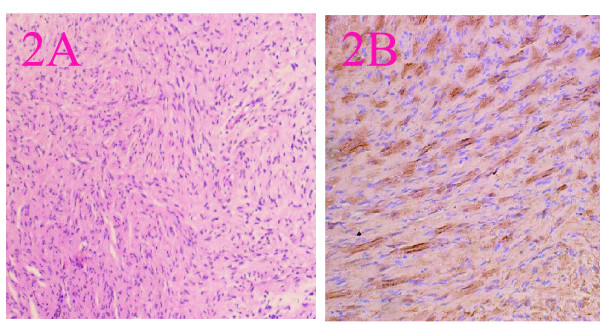
**Pathology features: A: Photomicrograph of nasal schwannoma showing cellular Antoni A and Antoni B areas (haematoxylin and eosin, original magnification × 20).B**: S-100 positive(Envision^™^ stain, original magnification × 40).

**Figure 3:**
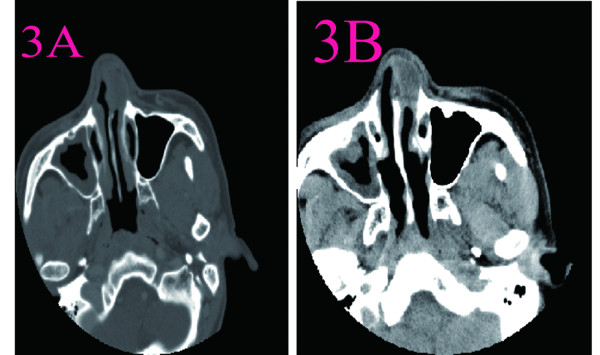
**CT scans of nasal cavity in Case 2.A**: CT imaging showed that a well-defined round soft tissue mass without central in the right nasal vestibule. The mass of density was uneven(20 ~ 36Hu). **B**: There was patchy enhancement on contrast CT.

**Figure 4:**
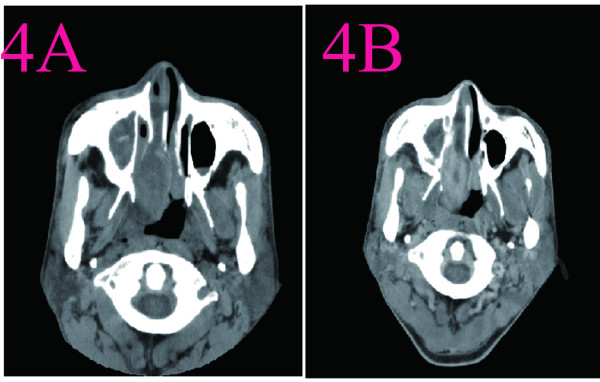
**CT scans of nasal cavity in Case 3.A**: CT scan showed that a well-defined large expansile soft-tissue mass in the left nasal cavity from choana to the inferior turbinate. **B**: There was patchy enhancement on contrast CT.

**Figure 5:**
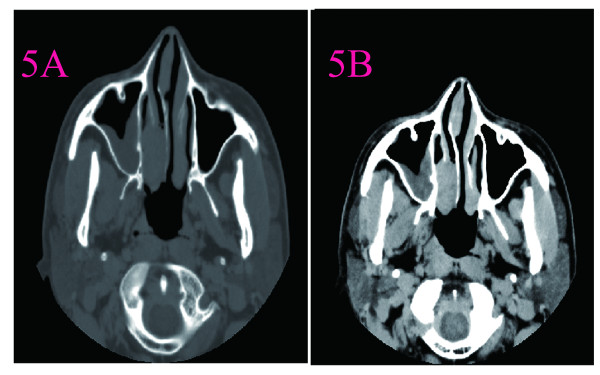
**CT scans of nasal cavity in Case 4. A**:CT scanning showed a 2.0 cm in diameter soft density mass that filling the left choana and extended into the nasopharynx. **B**: CT with contrast injection showed that the mass enhanceing inhomogeneously.

**Figure 6:**
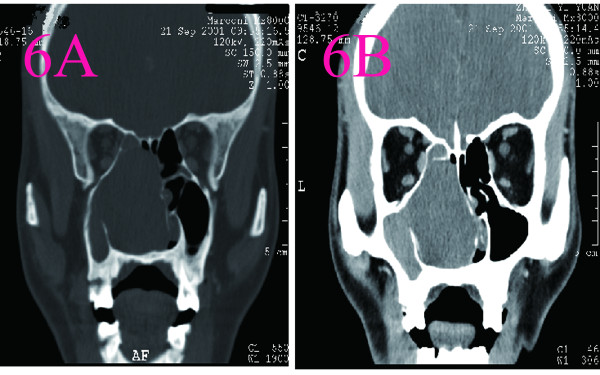
**CT scans of nasal cavity in Case 5. A**:A well-defined, inhomogeneous, 1.0 cm × 1.0cm soft mass that located at the anterior of the left nasal vestibule was observed by CT scan. The mass had an attenuation number of 17 (Hu). **B**:There was homogeneous enhancement on contrast CT(50Hu).

## Discussion

Neurilemmomas are usually composed of Schwann cells and are consequently also called schwannomas. Neurilemmomas can basically develop anywhere in the body, arising from the myelin sheaths of peripheral motor, sensory, sympathetic, and cranial nerves. The optic and olfactory cranial nerves are not potential sites of origin because they lack sheaths that contain Schwann cells [3]. The most common neurilemmoma in the head and neck is an acoustic neuroma arising from the eighth cranial nerve [4]. Only 4% involve the nasal cavity and paranasal sinuses. Neurilemmomas are predominantly benign, and the English literature only contains isolated cases of malignant schwannomas [5,6]. Tumor incidence in the sinonasal tract is not related to sex or age, with cases occurring in children and the elderly[4,7-15].In our present study, all five cases occurred in the nasal cavity, with two in the nasal vestibule and three in the nasal septum. The female/male ratio was 2:3, with an age range from 27 to 59 years and a median age of 48 years. Localization to the nasal septum and the nasal vestibule is exceedingly rare. The occurrence in the nasal septum is rare, with only 18 reported cases in the English literature. Malignant neurilemmoma of the nasal septum is extremely rare [8].

The clinical symptomatology is varied and nonspecific. The signs and symptoms usually depend upon the location or size of the tumor and subsequent involvement of surrounding structures, often in relation to signs of chronic nasal obstruction, such as rhinorrhea, epistaxis, and anosmia, and facial swelling [10]. Exophthalmos, facial swelling, and epiphora are less frequently observed [11]. In our present cases, the main symptom was nasal obstruction; only one other compliant, nasal bleeding, was recorded. All tumors were limited to the nasal cavity. However, these masses were easily misdiagnosed as other tumors, such as an inverted papilloma.

In general, benign neurilemmomas are well differentiated by a characteristic capsule derived from perineural cells in other locations. However, some authors have reported that neurilemmomas in the nasal cavity have been described as nonencapsulated masses [4,12,13]. These authors have suggested that this peculiarity could be explained by the development of these tumors from sinonasal mucosa autonomic nervous system fibers, which are devoid of perineural cells, similar to the case of gastric neurilemmoma. The absence of a capsule could be responsible for the lack of a cleavage plane and thus complicates surgical resection [14]. In our cases, however, we observed encapsulated tumors in four cases; only one case had a nonencapsulated tumor. Consequently, our cases were not consistent with the authors’ speculation. From our cases, we suggest that the lack of encapsulation does not imply malignancy.

Microscopically, neurilemmomas typically exhibit a biphasic histologic pattern of Antoni A and Antoni B areas. Antoni A areas are regions of high cellularity with spindle-shaped cells; the cells are often arranged in bundles, palisades, or whirls. Groups of compact parallel nuclei are also seen and are known as “Verocay bodies.” Antoni B areas are less cellular and do not exhibit a distinctive pattern. Additionally, neurilemmomas usually show intense immunostaining for S100 (particularly Antoni A areas), which may help to distinguish peripheral nerve sheath neoplasms from other tumors [16].We noted that the five benign nasal neurilemmomas had a mixed appearance, with Antoni A and B areas intermingled. A benign neurilemmoma of the paranasal sinuses should be differentiated from a malignant peripheral nerve sheath tumor, which is rarely described in this location.

Some findings have been reported on the special CT manifestations of neurilemmomas at other sites due to the varied pathologic changes [16,17]. In general, Antoni A and collagen have been attributed to high density areas on CT. Antoni B, obsolete bleeding, and cystic changes have been attributed to areas of low density on CT. From this set of data and an analysis of the literature, we suggest that nasal cavity neurilemmomas have the following characteristics [3,18,19]:

(1) Neurilemmomas tend to be solitary and are usually well-circumscribed tumors with an oval, round, or fusiform shape in the unilateral nasal cavity because these soft-tissue masses expand along peripheral nerves.

(2) The lesions usually have a mottled central lucency with peripheral intensification on contrast-enhanced CT scans. The heterogeneous appearance is related to areas of increased vascularity with adjacent non-enhancing cystic or necrotic regions [20].

(3) CT clearly depicts the relationship of the lesion to the surrounding bony structures; erosion is more common with large neurilemmomas. Bone destruction can be secondary to benign pressure erosion, and therefore such a finding should not necessarily indicate malignancy [20,21]. Because they tend to grow slowly, radiological studies typically show expansive soft-tissue masses along with the preservation of most bony margins. This finding can be helpful in differentiating neurilemmomas from malignancies, as the latter tend to be aggressive and destroy bone [3], but intracranial extension has also been reported [19,22]. Enlargement of neurilemmomas can lead to areas of cystic degeneration as the tumors outgrow their blood supply. Other common regressive changes are necrosis, lipidization, and formation of angiomatous clusters of blood vessels with focal thrombosis. The changes justify the adjectives that are sometimes used for these tumors.

As documented by previous reports [1-4] and also by our observations, nasal neurilemmoma commonly displays a soft-tissue mass without any distinctive features; therefore, the differential diagnosis includes a broad spectrum of lesions ranging from a neurofibroma cyst or hemangioma to malignant tumors such as melanoma and olfactory neuroblastoma.

In our present series, Case 3 was misdiagnosed as a nasal inverted papilloma. Cases 1,2, 4 and 5 were diagnosed as benign nasal tumors.

Differentiating between a neurilemmoma and neurofibroma can be particularly difficult because of the presence of overlapping histological features. Basically, neurilemmomas are often solitary and tender, with degenerative changes. Neurofibromas are more frequently multiple and associated with Von Recklinghausen’s disease; they are usually non-tender and less commonly present regressive changes. Neurofibromas are not encapsulated and are formed by a combined proliferation of all the elements of a peripheral nerve: axons, Schwann cells, fibroblasts, and probably perineural cells [23]. Malignant transformation of a neurilemmoma is exceedingly rare, but the risk increases in patients with Von Recklinghausen’s disease in whom the incidence of malignant transformation is about 10–15% [24]. On CT, neurofibromas show unclear boundaries or mild infiltration.

## Conclusions

Neurilemmoma should be considered in the differential of unusual nasal masses. Certain clinical and CT patterns (i.e., a mottled central lucency with peripheral intensification on contrast-enhanced CT scans) may be of use in the differential diagnosis.

## Competing interests

The authors declare that they have no competing interests.

## Authors’ contributions

JH designed the manuscript. Y-YB collected the materials. K-JC aided in the surgery and collected the materials. S-HZ performed the surgery and wrote the manuscript. L-XR analysed the imaging of CT. Z-JZ performed the immunohistochemical staining and analyzed the results. All authors read and approved the final manuscript.
